# Protocol for national mental health guidelines for community sport in Australia

**DOI:** 10.1136/bmjsem-2022-001426

**Published:** 2022-11-21

**Authors:** Caitlin Liddelow, Matthew J Schweickle, Jordan T Sutcliffe, Christian Swann, Richard Keegan, Simon Rice, Anthony David Okely, Stewart A Vella

**Affiliations:** 1 Global Alliance for Mental Health and Sport, School of Psychology, University of Wollongong, Wollongong, New South Wales, Australia; 2 Physical Activity, Sport and Exercise Research Theme, Faculty of Health, Southern Cross University, Coffs Harbour, New South Wales, Australia; 3 Research Institute for Sport and Exercise Psychology, Faculty of Health, University of Canberra, Canberra, Australian Capital Territory, Australia; 4 Centre for Youth Mental Health, Faculty of Medicine, Dentistry and Health Sciences, The University of Melbourne, Melbourne, Victoria, Australia; 5 Elite Sports and Mental Health, Orygen, Melbourne, Victoria, Australia; 6 Early Start, School of Education, University of Wollongong, Wollongong, New South Wales, Australia

**Keywords:** community, well-being, sport, sporting organisation

## Abstract

Organised sports are the most common settings for sports participation. Despite a range of documented benefits from participation, these positive outcomes are not always guaranteed. Emotional distress from pressure and injuries can mean some participants experience negative outcomes. To ensure organised sports are well equipped to promote the mental health of their members, evidence-based guidelines for them are required. Using a Community-Based Participatory Research framework, mental health guidelines for community sport will be developed. In Phase One, community sport stakeholders will participate in focus groups. The aim is to understand their preferences of the content, purpose and scope of the guidelines. In Phase Two, an e-Delphi study will be conducted with experts in mental health and sport in Australia to gather recommendations on the purpose and scope of the guidelines. In Phase Three, a national consensus meeting with an Expert Guideline Development Committee will be held to draft the guidelines. In Phase Four, follow-up focus groups will be held with community sport stakeholders to understand the usability and acceptability of the draft guidelines. In Phase Five, a second e-Delphi study will be conducted to provide feedback on the revised guidelines after community stakeholder review. In Phase Six, implementation case studies will assess the implementation of the guidelines in community sport clubs. These mental health guidelines will answer an urgent call for action by experts. The guidelines will be based on sector needs and preferences, be acceptable and useable, and be able to be implemented by community sport clubs globally by 2025.

**Key messages**

**What is already known on this topic**

- Over half of Australians participate in organised sport each year, however the psychological benefits of sport engagement are not always guaranteed. Despite this, there are currently no known mental health guidelines that community sport organisations can follow to ensure a psychologically safe environment.

**What this study adds**

- In collaboration with experts in the field and community stakeholders, acceptable and usable mental health guidelines for community sport will be developed, implemented and evaluated.

**How this study might affect research, practice or policy**

- The guidelines will fill an important gap by being the first-known national guide for best practice principles to support psychological safety and well-being in community sport.

## Background

Globally, around 40% of children and adolescents, and 20% of adults regularly participate in organised recreational sports.^1^ In Australia, organised sports clubs are the most popular setting for sports participation.^2^ National data suggests that over half of Australians (57.9%; 11.7 million) and nearly two-thirds of children participate in organised sport at least once per year.^3^ Such high participation rates demonstrate the wide reach of organised sport and underline its important context for the health and development of individuals globally.

There is a significant body of evidence that supports a range of physical and psychosocial benefits from engaging in organised sport, such as reducing the incidence of chronic disease, obesity and early death.^4^ Further, under the right circumstances, sport can be beneficial for one’s social interactions, peer networks, self-esteem, general well-being and various indicators of mental health.^5^ Sports participation can also protect against mental health problems among children and adolescents, such as depression and anxiety.^6^ Nevertheless, such positive outcomes are not guaranteed. For example, a landmark study from the USA showed that participation in particular forms of organised sports (eg, individual sports) was associated with greater incidence of mental health problems.^7^ More specifically, compared with those who did not participate in sport, participation in individual sports such as dance, tennis and martial arts was associated with higher levels of anxiety and depression, and more social difficulties.^7^ It is important, therefore, that deliberate efforts are made to ensure sports environments provide psychosocial benefits for all participants.

Recently, the pernicious effects of sport culture on participant mental health, particularly in elite sports, have received increased attention from academics and policy-makers alike. However, the emotional distress that results from injuries, pressure, bullying, overtraining and the competitive nature of sport has all been suggested to potentially negatively influence the mental health of participants, at both the elite^8^ and community/recreational levels.^9^ To protect against such effects, community sports organisations have an obligation to provide a psychologically safe environment for all involved.^10^ Psychological safety in sport has been defined as ‘the perception that one is protected from, or unlikely to be at risk of, psychological harm in sport’.^11^ Research with athletes, coaches and parents has reported that sports organisations play an important role in ensuring a psychologically safe environment.^12^ However, an audit of Australian community sports organisations revealed that although many identified mental health and well-being as important, no clear strategies for ensuring the psychological safety of their members were offered.^13^

Despite the growing consensus from stakeholders around the need for psychologically safe sports environments, there are currently no known published position statements, policies, or guidelines on mental health in recreational organised sports.^10^ This absence is particularly concerning given around 10% of the Australian population are likely to participate in organised community sports while experiencing a mental health problem.^14^ As of 2020, there were 13 position statements or guidelines related to mental health and sport endorsed by major sports organisations or governing bodies such as the International Society of Sport Psychology or the International Olympic Committee.^15^ The vast majority of these statements were concerned with the mental health of elite athletes, even though recreational athletes comprise more than 94% of all sports participants.^10^ There is a need to develop tangible guidelines for community sports organisations on how to protect and promote mental health for all involved in those organisations.^10^

### Research aims

To ensure recreational sports organisations are well equipped to protect and promote the mental health and well-being of their members and leaders, evidence-based guidelines are required. This project aims to create and assess the feasibility of implementing evidence-based mental health guidelines for the recreational sports sector. Importantly, these guidelines must be (a) based on sector needs and preferences, (b) acceptable and usable and (c) easily implemented by community sports clubs.^16^ To achieve these aims, three guiding research questions will be answered:

1. What are community sports stakeholder preferences for the content, purpose and scope of mental health guidelines?
2. What are stakeholders’ perceptions of the acceptability, usability, communication and monitoring of mental health guidelines in sport?
3. Are the implementation strategies of sports clubs and organisations effective? Why, or why not?

## Methods

### Framework

The development of mental health guidelines for the recreational sports sector will follow the Community-Based Participatory Research (CBPR) framework.^17^ The CBPR approach aims to combine knowledge and action to achieve societal change to improve health and health-related outcomes related to a topic of importance in the community. This framework is a collaborative approach to research that recognises the importance and uniqueness of all stakeholders associated with the project, and as such ensures the inclusiveness of these stakeholders throughout the research process.^17^ It aims to ensure that the community which is affected by the health issue has the opportunity to participate and make decisions in the project. More specifically, the researchers and community members work together to define the research methods, conduct the research and then translate and apply the findings. The co-design of this research project allows for both researchers and stakeholders to become engaged in each other’s activities and can thereby strengthen the sustainability of projects in the community. This framework has been successfully applied in other projects, such as in the development of a sport-based mental health literacy programme for adolescent males, Ahead of the Game.^12^

### Guideline development process

**Figure 1:**
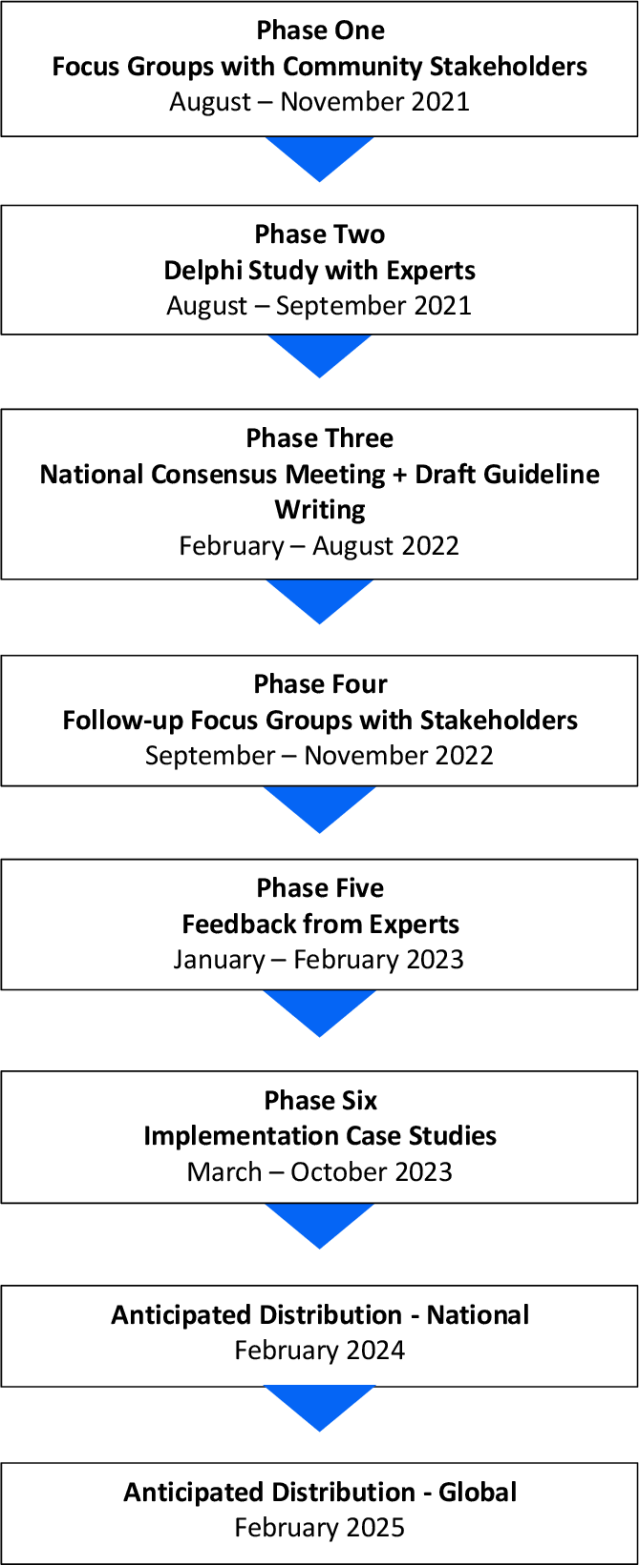
Proposed study timeline.

This guideline development process will follow the recommendations and procedures used in the development of the Australian physical activity guidelines,^18^ and the Australian National Health and Medical Research Council Guidelines for Guidelines,^16^ Grading of Recommendations, Assessment, Development and Evaluations^19^ and Appraisal of Guidelines Research and Evaluation^20^ guideline development resources. See figure 1 for a proposed study timeline.

### Patient and public involvement

In line with the CBPR framework, six phases involving the end users of the guidelines (ie, community sport club stakeholders) and experts in the area of mental health and sport in Australia will be used to answer the research questions and develop the guidelines. Community sport stakeholders will be involved throughout the development of the guidelines (Phases One and Four) and the implementation and evaluation of the guidelines (Phase Six).

### Phase One: focus groups with community stakeholders

To address the first and second research questions, we will explore the preferences of community sports stakeholders for the content and scope of mental health guidelines for community sports.

#### Participants and sampling

We will use a purposive sampling strategy^21^ to recruit participants to focus groups of between four and six participants each. Smaller sized focus groups are said to generate a richer discussion as well as being easier to manage.^21^ The participants will be groups of key stakeholders in community sport, such as players, parents, coaches, officials and committee members. In line with the broader aim of the project, we will recruit from all these different stakeholder groups to ensure broad representation among key stakeholders. Similarly, to ensure we have an in-depth understanding of community sports stakeholder preferences, we will sample from a diverse range of sports, locations, resources and mental health experiences, where possible. More specifically, participants will be recruited from around Australia, both from metropolitan and regional areas. Sport clubs that have had first-hand experience with mental health problems (eg, perhaps through the death of a current member), clubs that are open in their promotion of mental health and well-being and sport clubs that have not openly discussed experiences, will be purposely sampled.

Potential participants will be identified through the networks of the research team, as well as through publicly available information (eg, sport club websites and social media). The lead researcher will make initial contact with a key stakeholder from a sport club (eg, president) via email. This email will outline the research team, the aims of the project and what participation will involve. A participant information sheet will also accompany this email. This key stakeholder will then be encouraged to spread the word among their club. Interested participants are prompted to send their contact details to the lead researcher so they can be followed up for participation. Informed consent will be provided by all participants prior to participation in the focus group.

#### Methods

All focus groups will be held virtually via video conference due to COVID-19 health protocols and will be audio recorded. Focus groups will be scheduled at a convenient time for participants in each group. It is anticipated that each focus group will last between 45 min and 60 min and will be facilitated by two members of the research team. To counteract the potential drawbacks of virtual interviewing, rapport will be built with the participants beforehand by discussing the current sport season (eg, their performance thus far and upcoming games) and making light, general conversation with participants. The interviewers will introduce the project and its aims, and their backgrounds and allow the opportunity for questions before beginning the more formal discussion.^21^

A semi-structured interview schedule has been developed by the research team for this phase to guide the discussion and allow participants to elaborate on areas of perceived importance. The discussion will focus on four main areas: (a) the need for the proposed guidelines; (b) the scope of proposed guidelines; (c) the purpose of the proposed guidelines and (d) the implementation context (see online supplemental material). All recorded focus groups will be transcribed verbatim. Appraisal of the appropriate number of focus groups to conduct will be guided by the concept of information power^22^; however, we anticipate at least 10 diverse focus groups.

10.1136/bmjsem-2022-001426.supp1 Supplementary data

#### Analysis

Following transcription, data will be analysed using reflexive thematic analysis.^23^ To enhance trustworthiness of the account (ie, the methodological credibility), the research team will engage in peer debriefing through the use of both regular formal and informal meetings. In this process, the research team members will act as ‘critical friends’ by encouraging reflection on, and exploration of, alternative interpretations of the data. We will develop an audit trail that will document the decisions made throughout data collection and analysis.

### Phase Two: Delphi study with experts

To complement Phase One, a Delphi study with experts in Australia in the area of mental health and sport will be conducted. The Delphi technique is a research process, whereby questionnaires are sent out in several rounds to experts in the field until consensus on a topic is reached.^24^ This Delphi study will focus on synthesising expert opinions and guidance on what the scope and content of mental health guidelines in sport should be, as well as the role of community, state, and national sports organisations, should be in mental health prevention, promotion and care.

#### Methods and analysis

The Delphi process will follow the steps outlined by Keeney and colleagues^25^ for health research. We will also follow considerations and steps outlined by Shortt *et al*^26^ who used an ‘e-Delphi approach’. For example, the classical Delphi process contains four rounds,^27^ whereas the modified process allows for flexibility in the number of rounds required to reach consensus. Similarly, classical Delphi studies are conducted in person, however, the use of an 'e-Delphi approach' (ie, a Delphi study conducted online, typically anonymously) is becoming a more commonly used alternative with the increased use of technology.^27^ In line with the above, this Delphi study will use a sequential mixed-methods design whereby the data collection and analysis of each round informs the data collection and analysis of the subsequent round. The Delphi study will have a minimum of two rounds, but we do not anticipate more than four rounds. The agreed-upon consensus level for this Delphi will be 80%, following the recommendations from Keeney and colleagues^25^ who suggest a consensus level of between 70% and 80%.

#### Round one

To obtain experts’ opinions on the role of sports organisations in mental health protection, promotion and care, an online form using Qualtrics will be created. The form will ask the participants to respond to six open-ended questions related to mental health protection, promotion and care (eg, ‘In your opinion, what are the major considerations in providing mental healthcare to those involved in non-elite/recreational sport? Please elaborate on why.’), and whom should be responsible for each (eg, ‘Which sport bodies (eg, community clubs, local associations, state sport bodies and national sport bodies) should be responsible for mental healthcare in sport and what should their role entail?”).

Once all responses have been obtained, we will analyse the data qualitatively, with team members acting as critical friends throughout the data analysis process. We will apply content analysis, whereby statements that have the same or similar explicit meaning are grouped. This will be done for each of the six open-ended questions. When similar statements have been grouped, the research team will meet to discuss the findings and decide whether each group of statements can be collapsed into a single statement, or whether there are multiple statements required. Any individual statements (ie, statements that were not grouped) will be kept the same. We will group the final list of statements from this round into categories according to the initial question (eg, whether it fits with mental health protection, promotion or care), and be checked by the other authors to ensure the final statements reflect the meaning of the raw data.

#### Round two onwards

The minimum requirement for a Delphi study is two rounds.^25^ Following the qualitative data collected in round one, the second round and any rounds thereafter will adopt a quantitative approach to reaching consensus. Participants will first be presented with a written overview of the findings from the first round of data collection. After this, participants will be asked to rate each of the statements on two 5-point Likert scales, one on how much they agree with the statement (eg, strongly agree to strongly disagree) and the other related to the importance of the statement for the guidelines (eg, very important to not important at all). We will include an open-ended textbox to allow for any further comments or considerations to be entered.

Once all responses have been collected, quantitative analysis in the form of frequencies and descriptive statistics will be conducted in SPSS (v.26). Any statement that reaches 80% consensus, which is defined as 80% of participants selecting ‘strongly agree/agree’ or ‘very important/important’ on each scale, will be accepted as having reached consensus and excluded from the next round. Any statements that do not reach the agreed-upon consensus level will be collated and form part of the next round. As part of the next round, participants will also be provided with a summary of the previous round outlining which statements achieved consensus. This process will continue until a predetermined sufficient number of statements/items reach consensus. Typically, three rounds are appropriate for most Delphi studies.^25^

### Phase Three: national consensus meeting

Following stakeholder engagement and expert opinion, we will synthesise the evidence from those two phases with other research in the area, to develop draft guidelines. This is the final phase in addressing research question one.

#### Expert guideline development committee

We will establish a guideline development committee to ensure the guidelines meet the requirements of guideline development, as well as meet the needs of stakeholders. The role of the committee is to engage in the overall development of the guidelines (see Phase Three for more information). The committee will include a diverse range of representatives from key expert stakeholders in mental health and sport around Australia. These include Sport Medicine Australia, Sport Australia, Football Australia, the Australian Football League and the Australian Psychological Society. The committee will also contain expert clinicians and researchers in the area of mental health, and sport and exercise psychology. There is no preference for the size of the committee; instead, the focus will be on ensuring a breadth of representation, expertise in mental health and sport and experience in guideline development. Community sport stakeholders will not be part of this committee.

#### Participants

The expert guideline development committee, and the other members of the research team, will be invited to participate.

#### Methods

The evidence from Phase One (focus groups with stakeholders), Phase Two (Delphi study with experts) and other literature written by the research team or experts on the committee that is not explicitly part of this project, but is related to the project aims (ie, systematic review on mental health interventions in non-elite sport,^28^ and a meta-synthesis of existing position statements on mental health in sport^15^), will be synthesised by the research team. All members of the guideline development committee, along with the research team, will meet in a mutually convenient location to consider all the evidence to support guideline development. The committee will meet for 2 days and collaboratively: (a) consider the evidence for guideline development and ensure that the perspectives of end-user groups from Phase One are integrated; (b) discuss, refine and finalise the scope and purpose of the guidelines and (c) develop a set of draft guidelines. Each guideline will be put to a vote where members of the guideline development committee can cast a vote in real-time for whether (a) a specific area of interest (eg, coach education) should form part of the guidelines and if so, (b) what the draft guideline should be, based on the evidence. For an area of interest and its associated guideline to reach consensus, a minimum of 80% agreement will be required. Areas or guidelines that do not reach consensus will be discussed and amended, if appropriate. If any area or guideline does not reach 80% consensus from the expert guideline development committee, it will be excluded from the draft guidelines. Two members of the research team will act as ‘scribes’ during this meeting to capture the breadth and details of conversations and decision-making.

### Phase Four: follow-up focus groups with stakeholders

To answer research question two, follow-up focus groups with community sports clubs and local and national sports organisations in Australia will be conducted to explore their perceptions of the draft guidelines. As used in Phase One, the CBPR framework will be adopted.

#### Participants and sampling

We will use a purposive sampling procedure^21^ in this phase to understand the acceptability, feasibility and usability of the draft guidelines. We will re-contact the sports clubs that participated in the first round of focus groups and invite them to participate in a follow-up focus group. This will enable us to ensure we have understood and met the needs that were expressed in Phase One. We will also approach clubs and sports organisations that did not participate in Phase One. We will target clubs and sports that will provide varied perspectives on the draft guidelines. By targeting local (eg, regional sport associations) and national (eg, Sport Australia) sports organisations, we hope to understand the preferences for implementation and monitoring processes at these different levels of sport management. Clubs in varying geographical locations (eg, from around Australia, in metropolitan and regional areas) with varying gender ratios, number of members and club resources will be targeted using publicly available information and contact details. All previous clubs, new clubs and organisations will be approached via email, and all participants in the focus groups will receive a $20 retail voucher as compensation for their time. In line with Phase One, the number of focus groups conducted will be guided by information power,^22^ and the size of each focus group will be between four and six participants, consistent with the size of groups in Phase One.

#### Methods

As in Phase One, focus groups will be moderated by two members of the research team using semi-structured interview guides. However, the discussion will be focused on: the acceptability of the guidelines (eg, ‘What do you think of the guidelines?’); usability of the guidelines (eg, ‘How would your club implement the guidelines?’); communication of the guidelines (eg, ‘How do you think the guidelines should be communicated to clubs?’) and monitoring of the guidelines (eg, ‘In your club, who should be responsible for ensuring the guidelines are implemented?’).

#### Analysis

To understand stakeholders’ views and perceptions on the acceptability, feasibility and usability of the draft guidelines, reflexive thematic analysis will be used to analyse and identify patterns in these data,^23^ as outlined in Phase One.

### Phase Five: feedback from experts

Following stakeholder feedback in Phase Four, we will reconvene the Guideline Development Committee via a Delphi study to consider the feedback on the draft guidelines.

#### Methods and analysis

The same Delphi process outlined in Phase Two will be used for this phase. A sequential mixed-methods design will be used, such that the data collection and analysis of each round informs the subsequent round. Similar to Phase Two, expert panel members will be presented with proposed changes to the draft guidelines following stakeholder feedback and asked to quantitatively rate how much they agree with each change on a 5-point Likert scale (semantic anchors ranging from strongly agree to strongly disagree). They will be offered the opportunity to offer qualitative feedback on each change. The final section will ask participants to rank what order they believe each of the guidelines should be presented. The same process and analysis as outlined in Phase Two will be used here to determine consensus.

### Phase Six: implementation case studies

The final phase of developing and refining the guidelines is to undertake implementation case studies with community sports clubs. In this phase, we aim to evaluate the experiences, barriers, constraints and opportunities for the implementation of the mental health guidelines. This final phase addresses our final research question (ie, are the implementation strategies of sports clubs and organisations effective? Why, or why not?).

#### Participants and sampling

The research team will liaise with key industry stakeholders, such as local and national sports organisations, to identify sports clubs whereby the guidelines and their implementation are likely to be influenced by contextual factors (eg, geographic location, club resources, gender ratio and membership size). This purposive sampling strategy will ensure the implementation of the guidelines is assessed across a range of contexts. We anticipate that we will recruit approximately 10 sports clubs with whom the research team will work closely to implement the guidelines.

#### Methods

As per the case study approach outlined by Stake,^29^ we will use multiple data sources and data collection methods. First, each club will be asked to record all implementation activities and their impacts in an activity diary. They will also have the opportunity to reflect and provide feedback on components of the guidelines that require improvement or refinement through one-on-one interviews during the implementation phase. At the end of the sports season, all club members who have been involved in the implementation of the guidelines will be invited to participate in a focus group. The focus group will cover: (a) the individual experiences of implementing the guidelines and (b) the effectiveness of their implementation efforts. The use of multiple sources of evidence can increase the internal validity of a study while also capturing varying responses and views to the same questions to grasp a more holistic understanding of the implementation of the guidelines.^29^

#### Analysis

For qualitative data, the same analysis and trustworthiness procedures as used in Phases One and Four will be used. The multiple data sources will be triangulated for combined data analysis.^29^ The variations within each case study (ie, within-case) and the differences between case studies (ie, between-case) regarding the relationships between the implementation strategies and the outcomes will be noted.

## Discussion

This study uses a multi-phase, CBPR-based framework to develop, implement and evaluate mental health guidelines for organised recreational sports. This study answers a call for action^10^ to equip recreational sports organisations with the necessary guidelines for ensuring a psychologically safe environment. These guidelines will be developed collaboratively with both experts in the field and community stakeholders at all levels (ie, national, state and community sports bodies and organisations). Through six phases of research, the final guidelines will be based on sector needs and preferences, be acceptable and useable, and be able to be implemented by community sports clubs. The guidelines will fill an important gap, both in research and policy, by being the first-known national guide for best practice principles to support psychological safety and well-being in community sport.

When considering the potential limitations of this study, it is noted that self-selection bias may occur. Although a range of clubs that are contextually and theoretically varied will be approached for participation, clubs that are already actively involved with mental health and well-being promotion or programmes, or deem it as a priority, may be more willing to participate in the focus groups and implementation case studies. While it is important to gather perspectives from all contextual settings, clubs that do not view well-being as a priority may be less likely to participate and thus the needs of these clubs may not be adequately captured. Despite this, our community-based approach has strengths. Through collaborating and ensuring community involvement throughout the development of the guidelines, there may be greater levels of community engagement and ownership when implementing the guidelines in the final phase.^30^ It is hoped this engagement throughout will lead to greater acceptance and implementation beyond the length of the project.

One risk to the fidelity of the project, and the final implementation of the guidelines, is the nature of many sports clubs being run by volunteers who often lack time to participate in further activities outside of the club. To overcome this potential risk, we aim to ensure that community and stakeholder participation in any phase of the guideline development does not require significant additional time or effort on the participant’s behalf. For example, we will hold focus groups at a mutually convenient time and aim to ensure the guidelines are easy to understand, implement and monitor and require minimal resources.

## Data Availability

No data are available.
