# Supplementary material for: Protocol for national mental health guidelines for community sport in Australia
Source: BMJ Open Sport Exerc Med. 2022 Nov 21;8(4):e001426. doi: 10.1136/bmjsem-2022-001426 (PMC9680186; doi:10.1136/bmjsem-2022-001426)
Supplement: Supplementary data [file bmjsem-2022-001426supp001.pdf]

Developing mental health guidelines for sport: Perceptions of stakeholders.

Semi-structured interview guide

**Demographics**	
Age	
Sport	
Gender	
Type of competition	

Note: Participants will be probed based on their answers to the questions listed below.

Focus group questions

1) Do you think mental health guidelines are needed in sport? Why?
2) What do you think should be covered in mental health guidelines for sports clubs?
3) Is there anything in particular that shouldn't be covered in mental health guidelines for sports clubs?
4) What do you hope your club would achieve through guidelines for mental health?
5) What specific content would you like to see as part of the proposed guidelines?
6) What are the barriers to putting guidelines such as these into practice within your club?"
